# Sorting Nexin 10 Mediates Endosomal Acidification and Autophagy to Promote Influenza A Virus Infection

**DOI:** 10.3390/v18040460

**Published:** 2026-04-12

**Authors:** Lizhu Chen, Haobin Li, Huiyi Guo, Jinlong Liang, Yingyuan Zhong, Xucheng He, Wenjiao Wu, Shuwen Liu

**Affiliations:** 1 Guangdong Provincial Key Laboratory of New Drug Screening, School of Pharmaceutical Sciences, Southern Medical University, Guangzhou 510515, China; 2 State Key Laboratory of Organ Failure Research, Guangdong Provincial Institute of Nephrology, Southern Medical University, Guangzhou 510515, China; 3 Department of Pharmacy, The Affiliated Guangdong Second Provincial General Hospital of Jinan University, Guangzhou 510317, China; 4 MOE Key Laboratory of Infectious Diseases Research in South China, Southern Medical University, Guangzhou 510515, China; 5 NMPA Key Laboratory of Drug Metabolism Research and Evaluation, Pingshan Hospital, Southern Medical University, Shenzhen 518000, China

**Keywords:** Sorting Nexin 10, Influenza A Virus, endosome acidification, autophagy, ubiquitination

## Abstract

The infection cycle of the Influenza A Virus (IAV) typically requires host factors to regulate replication and proliferation. However, the roles of these factors remain undiscovered. This study focuses on Sorting Nexin 10 (SNX10), which is involved in regulating membrane trafficking and endosomal stabilization. Our previous study identified that SNX10 facilitates the replication of human coronavirus OC43 through enhancing clathrin-mediated endocytosis. In our present study, we found that SNX10 significantly promoted IAV infection in host cells. The conditional knockout of *Snx10* in mice lungs prolonged survival following IAV challenge. Mechanistically, SNX10 facilitated the production of acidic endosomal vesicles and promoted the accumulation of pro-viral autophagic structures, a process supported by the specific interaction between SNX10 and the viral NP and M2 protein of IAV. Blocking SNX10-mediated acidic endosomal vesicles and autophagosome formation demonstrated antiviral effects. Moreover, IAV infection increased SNX10 protein levels by suppressing its ubiquitination, suggesting that SNX10 could serve as a potential host-derived antiviral drug target.

## 1. Introduction

Influenza A Virus (IAV) is a major respiratory pathogen that poses a persistent threat to global public health [1]. As a member of the Orthomyxoviridae family, IAV contains a single-stranded, negative-sense RNA genome with eight segments encoding at least 22 viral proteins [2]. IAV is responsible for seasonal epidemics that result in an estimated 290,000 to 650,000 deaths annually worldwide [3]. IAVs are also the major agents behind influenza pandemics, underscoring their significant epidemiological impact.

Current strategies to control influenza rely on vaccines and antiviral drugs. There are four classes of drugs available for influenza: (a) matrix protein 2 (M2) ion channel inhibitors, such as amantadine and rimantadine (Symmetrel, Flumadine); (b) Neuraminidase inhibitors, including peramivir, zanamivir, laninamivir, and oseltamivir (Rapivab, Relenza, Inavir, Tamiflu); (c) RdRp inhibitors, like favipiravir (Avigan); and (d) PA inhibitors, such as baloxavir marboxil (Xofluza) [4]. Vaccination is the primary recommended preventive measure against seasonal influenza. However, the continuous antigenic drift and shift in IAV lead to the frequent mismatch between seasonal vaccines and circulating viral strains, which greatly reduces the protective efficacy of vaccines in clinical applications [5]. This inherent limitation of vaccine-based prevention strategies highlights the urgency of identifying novel host cellular factors as therapeutic targets for the development of broad-spectrum anti-IAV drugs.

The replication cycle of IAV is closely linked to host cellular processes. The virus initially binds to sialic acid receptors on the host cell via its membrane protein hemagglutinin (HA) [6]. Then the virus is internalized via clathrin-dependent endocytosis or clathrin-independent macropinocytosis. Subsequent endosomal acidification activates the M2 ion channel [7,8]. These early endosomes are transported to the perinuclear region by actin and microtubules [9]. They mature into late endosomes with a low-pH (approximately pH 5.0) by fusing with acidic lysosomes. This acidic environment induces a structural rearrangement of HA, mediating fusion between the viral and endosomal membranes [10]. The vacuolar-type H^+^-ATPase (v-ATPase) complex is essential for endosomal acidification, as it hydrolyzes ATP to provide the energy for pumping protons into the endosomal lumen [11]. Simultaneously, protons enter the virion through the M2 ion channel [12]. This leads to the release of viral ribonucleoproteins (vRNP), which are transported into the nucleus to initiate viral replication and translation. Finally, the assembled progeny virions are released from the infected cell to start a new round of infection.

As one of the smallest cellular parasites, IAV relies extensively on host cellular machinery to complete its replication cycle. To ensure successful infection, IAV strategically hijacks a variety of host pathways, reprogramming fundamental cellular processes to favor viral propagation. The systematic identification of host factors essential for IAV replication serves a dual purpose: it deciphers the molecular logic of viral–host interactions and unveils potential therapeutic targets for antiviral intervention. Indeed, several therapeutic strategies have already been developed based on this principle. For instance, the sialic acid analog DAS181 blocks viral attachment to host cells by cleaving sialic acid residues from the cell surface [13], while dynasore inhibits clathrin-mediated endocytosis by suppressing dynamin activity, thereby preventing viral entry [14]. These examples underscore the therapeutic potential of targeting host pathways co-opted by IAV.

Among the host processes manipulated by IAV, autophagy has emerged as a particularly complex and intriguing target. IAV structural proteins—including nucleoprotein (NP) and M2—have been shown to induce autophagosome accumulation via subversion of the AKT-mTOR signaling axis. This manipulation creates membranous vesicular platforms that facilitate viral entry and vRNA synthesis [15]. In addition, M2 disrupts the TBC1D5-Rab7 complex, a key regulator of late endosomal trafficking, thereby protecting endocytosed virions from lysosomal degradation and promoting their trafficking to the plasma membrane to support viral budding [16]. However, the relationship between autophagy and IAV is highly context-dependent. Depending on the specific host cell environment and viral genetic background, autophagy can exert either pro-viral or antiviral effects. This duality has complicated our understanding of IAV-host interactions and emphasized the need for finer resolution of the spatial and temporal dynamics of autophagic regulation during infection.

SNX10, a member of sorting nexin (SNX) family proteins, is a phosphoinositide-binding adaptor protein involved in endosomal sorting and trafficking. It plays a critical role in maintaining endosome–lysosome homeostasis and function [17,18]. Dysregulation of SNX10 has been implicated in several diseases, including osteoporosis [17], inflammatory bowel disease [19], atherosclerosis [20], and cancer [21]. Mechanistically, SNX10 interacts with the v-ATPase complex to stimulate proton pumping and facilitate the formation of acidic intracellular compartments such as endosomes and lysosomes [18,22]. Notably, v-ATPase-mediated acidification is essential for the uncoating and entry of many enveloped viruses, including IAV [23]. Pharmacological inhibitors of v-ATPase, such as bafilomycin A1 and diphyllin, have been shown to effectively suppress IAV infection by preventing endosomal acidification [24]. Our previous work identified SNX10 as a host factor that facilitates the replication of human coronavirus OC43 (HCoV-OC43). Mechanistically, SNX10 directly interacts with the μ1 subunit of the adaptor protein complex 2 (AP2M1), promoting its phosphorylation and enhancing clathrin-mediated endocytosis. This interaction underscores a broader role for SNX10 in regulating endocytic pathways exploited by multiple viruses, suggesting a conserved mechanism of viral entry facilitation [25].

Based on these observations, we hypothesized that SNX10 may influence IAV infection by modulating the acidic endosomal environment crucial for viral entry. Here, we investigate the functional role of SNX10 in the context of IAV infection, with particular focus on its involvement in regulating autophagy and endosomal acidification. Through this work, we aim to elucidate the molecular mechanisms by which SNX10 facilitates or restricts IAV replication, thereby contributing to a deeper understanding of host–pathogen interactions and uncovering potential avenues for therapeutic intervention.

## 2. Materials and Methods

### 2.1. Cells and Viruses

Madin-Darby canine kidney (MDCK) cells, human lung epithelial (A549) cells, and mouse lung epithelial cells (MLE-12) were procured from the American Type Culture Collection (ATCC, Manassas, VA, USA). Cell culture was performed using Dulbecco’s Modified Eagle Medium (DMEM) supplemented with 10% fetal bovine serum (FBS) and 1% penicillin/streptomycin (Thermo Fisher Scientific, Waltham, MA, USA) for MDCK and MLE-12 cells, while A549 cells were maintained in RPMI 1640 medium containing 10% FBS and 1% penicillin/streptomycin. IAV H1N1 strains A/Puerto Rico/8/34 (PR8) and A/WSN/33 (WSN) were propagated through allantoic cavity inoculation of 10-day-old embryonated chicken eggs. Viral titers were determined using a standard plaque assay protocol.

### 2.2. Mice

*Snx10*^flox/flox (fl/fl)^ mice were provided by Prof. Xiaoyan Shen (Fudan University). Genotyping was performed by PCR using tail-derived DNA with primers: *Snx10*-F (5′-TGTGGGAGATGATAGCTCTGATTG-3′) and *Snx*10-R (5′-TGGCCTAGAACTTACTGCAACG-3′). The PCR protocol included 94 °C for 1 min, 35 cycles of 98 °C for 15 s, 60 °C for 30 s, 72 °C for 60 s, and a final extension at 72 °C for 5 min. Amplicon sizes were 321 bp for *Snx10*^fl/fl^, 243 bp for *Snx10*^−/−^, and both for *Snx10*^fl/−^. Lung epithelial-specific *Snx10* conditional knockout (cKO) mice were generated by crossing *Snx10*^fl/fl^ with *Sftpc*-CreERT2 mice, yielding wild-type (WT) (*Sftpc*-CreERT2^−/−^-*Snx10*^fl/fl^) and cKO (*Sftpc*-CreERT2^+/−^-*Snx10*^fl/fl^) lines. Mice were housed under standard conditions with free access to food and water. All procedures followed National Institutes of Health (NIH) guidelines and were approved by the Animal Ethics Committee of Southern Medical University. At 4 weeks of age, mice received tamoxifen (Sigma, St. Louis, MO, USA) intraperitoneally for 5 days, followed by 5 days of adaptation prior to infection.

### 2.3. In Vivo Antiviral and Anti-Inflammatory Evaluation

For survival analysis, WT and cKO mice were intranasally infected with PR8 (2LD50) and monitored for 14 days (*n* = 11 per group). Lung tissues were collected on day 5 post-infection to assess lung index (lung-to-body weight ratio). Viral load, and inflammatory cytokine mRNA levels were detected by quantitative reverse transcriptase-PCR (qRT-PCR). Histopathological changes were evaluated by H&E staining. Serum IFN-γ levels were measured by ELISA (MM-0182M1).

### 2.4. Antibodies and Compounds

Antibodies were used for immunoblotting and immunofluorescence assay as follows: rabbit monoclonal β-actin antibody (Cell Signaling Technology, Danvers, MA, USA, 4970S); goat polyclonal SNX10 antibody (Santa Cruz Biotechnology, Dallas, TX, USA, sc-104657); rabbit polyclonal SNX10 antibody (Abcam, Cambridge, UK, ab221593); mouse monoclonal NP antibody (Abcam, ab66191 for immunoblotting, ab20343 for immunofluorescence); rabbit polyclonal or mouse monoclonal FLAG antibody (Sigma-Aldrich, St. Louis, MO, USA, F7425 or F1804); Anti-HA tag (Cell Signaling Technology, 3724); rabbit IgG antibody (FITC) (GeneTex, Irvine, CA, USA, GTX77059); goat Anti-Mouse IgG H&L (FITC) ((Abcam, ab6785); HRP-conjugated goat anti-mouse or rabbit IgG (7076S and 7074S); HRP-conjugated rabbit anti-goat IgG (Fdbio science, Hangzhou, Zhejiang, China, FDG007). Hoechst 33258 (Sigma, 94403); Poly(I:C), Cycloheximide (CHX) (Sigma-Aldrich, 239765), MG-132 (Sigma-Aldrich, M8699), Neutral Red powder (Sigma-Aldrich, P3532) and Bafilomycin A1 (Sigma-Aldrich, B1793). LysoTracker Red DND-99 (Invitrogen, Carlsbad, CA, USA, L7528) was purchased from Thermo Fisher Scientific.

### 2.5. Cell Transfection

For overexpression assay, expression plasmid pCMV3-mSnx10-HA or negative control pCMV3-C-HA-NCV was purchased from Sino Biological, Beijing, China (MG51516-CY, CV013). MLE-12 cells were transfected with plasmid pCMV3-mSnx10-HA or negative control pCMV3-C-HA-NCV using Lipofectamine 3000 (Life Technologies, Carlsbad, CA, USA). A549 cells were transduced with lentivirus carrying human SNX10 (pCMV3-Snx10-Flag) or negative control (pCMV3-C-Flag), which were purchased from GeneCopoeia, Rockville, MD, USA (LPP-W0389-Lv121-100, LPP-NEG-LV121-025). To generate stable cell lines, after transfection for 24 h, the medium containing lentivirus was then replaced with RPMI 1640 complete medium with 2 μg/mL puromycin (Sigma-Aldrich, 540222) for 3 days. The overexpression efficiency of SNX10 was evaluated by Western blotting.

SNX10 small interfering RNA (siRNA) (5′-CAGGGCTTGGAAGATTTCCT CAGAA-3′) were synthesized by GenePharma, Suzhou, Jiangsu, China. For knockdown assay, A549 cells or MLE-12 cells were transfected with siRNA diluted in Opti-MEM (Life Technologies) using Lipofectamine 3000 (Life Technologies), following the manufacturer’s recommended transfection protocol. The knockdown effect on the SNX10 expression was confirmed by qRT-PCR and Western blotting.

### 2.6. Quantification of mRNA Expression

The qRT-PCR was used to measure the expression of specific genes. Total RNA was extracted with Trizol according to the manufacturer’s instructions and reverse transcribed to cDNA. Then the qRT-PCR was performed and analyzed by ABI 7500 system (Applied Biosystems, Foster, CA, USA). GAPDH mRNA expression was used to normalize the samples. All primers used for qRT-PCR analysis were designed for this study as follows:

*homo*-SNX10: AGACATTGAGGCGTGTGTTTC (forward),
*homo*-SNX10: TCCTGCGGAGCTGTATTTACTT (reverse),
*mus*-SNX10: TTCATTACATGGCTTGCCTCCT (forward),
*mus*-SNX10: CAGACACTCACGAACTCCTCTT (reverse),
*homo*-GAPDH: AGGGCAATGCCAGCCCCAGCG (forward),
*homo*-GAPDH: AGGCGTCGGAGGGCCCCCTC (reverse),
*mus*-IFN-β: CCTGGAGCAGCTGAATGGAA (forward),
*mus*-IFN-β: CCACCCAGTGCTGGAGAAAT (reverse),
*mus*-TNF-α: CAGGCGGTGCCTATGTCTC (forward),
*mus*-TNF-α: CGATCACCCCGAAGTTCAGTAG (reverse),
*mus*-IFN-γ: GCCACGGCACAGTCATTGA (forward),
*mus*-IFN-γ: TGCTGATGGCCTGATTGTCTT (reverse),
*mus*-IL10: GCCACGGCACAGTCATTGA (forward),
*mus*-IL10: ACCTGCTCCACTGCCTTGCT (reverse).

### 2.7. Virus Infection

After transfection, cells were washed twice with phosphate-buffered saline (PBS) and then incubated with IAV for 1 h. The inoculum was subsequently removed. After two washes with PBS, the cells were cultured with fresh RPMI 1640 serum-free medium, while MLE-12 cells were cultured in DMEM supplemented with 0.1% FBS and 1 μg/mL TPCK-trypsin. Supernatants and proteins were harvested at the indicated times.

### 2.8. Plaque Assay

The titer of the supernatant collected from influenza-infected cells was quantified using a plaque assay on MDCK cells. MDCK cells were washed twice and infected with serial dilutions of the samples at 37 °C for 1 h. The supernatant was then discarded and replaced with 3 mL of replacement media (1.5 mL 2 × DMEM and 1.5 mL avicel (1.2%) supplemented with 1.5 μg/mL trypsin, 0.5% BSA, 20 mM HEPES, and 1% penicillin–streptomycin). After infection for 72 h at 37 °C, cells were stained with 4% paraformaldehyde containing 2% crystal violet for 30 min at room temperature.

### 2.9. Western Blot Analysis

Total protein was extracted from cells using RIPA buffer containing protease and phosphatase inhibitors. Equal amounts of protein were separated by 10% SDS-PAGE and transferred onto PVDF membranes. The membranes were blocked with 5% skim milk in TBST for 1 h at room temperature and then incubated overnight at 4 °C with primary antibodies diluted in blocking buffer. After washing, membranes were incubated with HRP-conjugated secondary antibodies for 1 h at room temperature. Protein signals were visualized using an ECL substrate (Thermo Scientific) and detected with a chemiluminescence imaging system.

### 2.10. Immunofluorescence Assay

Cells were fixed with 4% paraformaldehyde for 20 min at room temperature, permeabilized with 0.1% Triton X-100 for 5 min, and blocked with 3% BSA for 1 h. They were then incubated with primary antibodies overnight at 4 °C, followed by incubation with fluorescently labeled secondary antibodies for 1 h at room temperature. Nuclei were counterstained with Hoechst 33258 for 10 min. Fluorescent images were acquired using a confocal laser scanning microscope (LSM 880, Carl Zeiss, Oberkochen, Germany) and processed with ZEN 2 (blue sdition) software (Carl Zeiss, Oberkochen, Germany).

### 2.11. Acidification Assay

Neutral Red uptake was used to evaluate acidic vacuole formation in A549 cells stably overexpressing SNX10 or empty vector. Cells cultured in six-well plates were incubated with 0.05% Neutral Red solution at 37 °C for 10 min. After imaging, cells were washed with cold PBS, and the dye was extracted with extraction solution for 10 min at room temperature. The absorbance of the extracted solution was measured at 540 nm using a microplate reader.

LysoTracker Red DND-99 staining was used to visualize acidic organelles [26]. For lysosomal staining, cells infected with IAV (MOI = 10) were incubated with 50 nM LysoTracker Red DND-99 in serum-free medium at 37 °C for 15 min, followed by nuclear staining with Hoechst 33342. Fluorescence images were captured using a confocal microscope.

### 2.12. Immunoprecipitation Assay

Immunoprecipitation assay was devised to determine the impact of influenza virus infection on the ubiquitination of SNX10. A549 cells stably expressing Flag-SNX10 were transfected with HA-ubiquitin for 24 h. Subsequently, the cells were infected with IAV (MOI = 1) for 20 h, followed by treatment with MG-132 for the final 4 h to stabilize ubiquitinated proteins. Cells were then lysed using NP-40 lysis buffer (1 M Hepes-NaOH pH 7.6, 0.5 M EDTA, 5 M NaCl, 2.5% NP-40 and protease inhibitor). After centrifugation, SDS was added into protein lysates to a final concentration of 1% and heated at 100 °C for 5 min. The lysates were subjected to immunoprecipitation overnight at 4 °C with an anti-Flag antibody conjugated to protein G-agarose beads. The immunoprecipitated complexes were eluted by boiling in SDS sample buffer and analyzed by Western blotting.

### 2.13. Statistical Analysis

Survival analysis was performed with Kaplan–Meier curves using GraphPad Prism 10.6.1 (Graphpad Software, San Diego, CA, USA), and statistical significance was assessed with the Log-rank test. For the remaining data, graphs were constructed in GraphPad Prism. The statistical significance of differences between relevant experimental groups was determined by a Student’s *t*-test. Significance was considered at *p* < 0.05, with *p*-values represented as follows: ns for no significance; * for *p* < 0.05; ** for *p* < 0.01; *** for *p* < 0.001; **** for *p* < 0.0001. All findings were verified in a minimum of three independent experimental replicates.

## 3. Results

### 3.1. SNX10 Deficiency Confers Protection Against IAV Challenge

**Figure 1:**
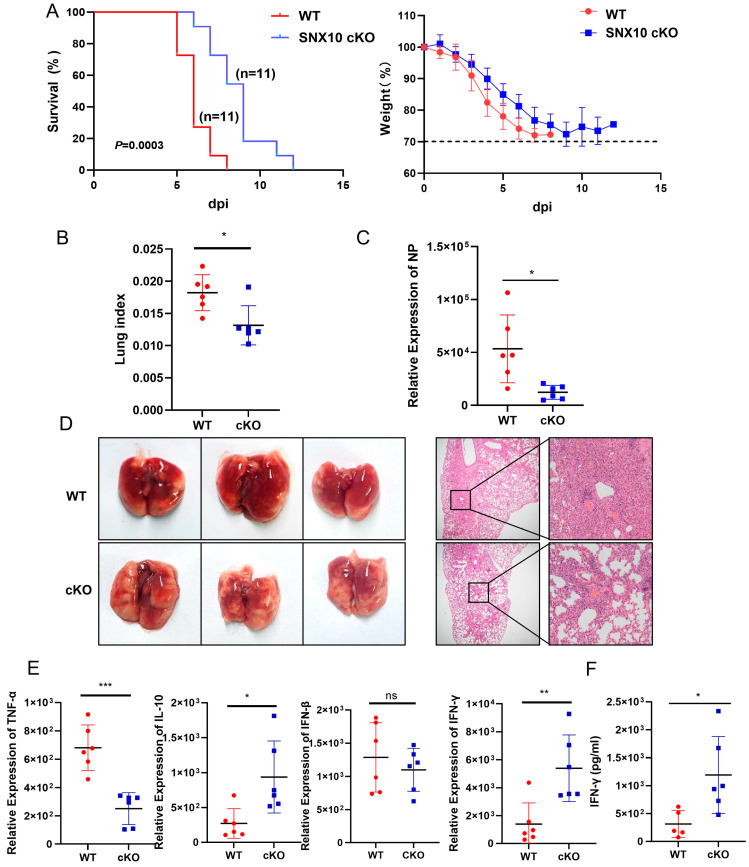
SNX10 deletion in lung protected mice from PR8 virus infection and reduces virus-induced inflammatory response. (**A**) Survival rates and body weight changes in mice (*n* = 11) were monitored daily for 14 days post-infection (dpi). (**B**) Lung index of mice sacrificed at 5 dpi; (**C**) qRT-PCR analysis of viral RNA expression. (**D**) Representative images of lung tissue morphology (H&E staining) at 5 dpi. Scale bars = 500 μm and 100 μm. (**E**) qRT-PCR analysis of TNF-α, IL-10, IFN-β, and IFN-γ mRNA expression. (**F**) ELISA assay of IFN-γ expression in the serum of IAV infected mice. Data are presented as the mean ± SD of three independent experiments and analyzed by two-tailed Student’s *t*-test (* *p* < 0.05, ** *p* < 0.01, *** *p* < 0.001).

To investigate the regulatory role of SNX10 in the context of IAV infection, we generated lung epithelium-specific conditional knockout (cKO) mice with efficient knockdown confirmed by immunoblotting (Figure S1A). Following intranasal challenge with a lethal dose (2 LD_50_) of IAV PR8 strain, both wild-type (WT) and cKO mice ultimately succumbed to infection. However, cKO mice exhibited significantly prolonged survival compared to WT controls (median survival: 13 days vs. 7 days; *p* = 0.00003) and experienced delayed and attenuated weight loss (Figure 1A). Furthermore, when we harvested the mice lungs at day 5 post-infection, cKO mice demonstrated reduced disease severity, as evidenced by a significantly lower lung index (lung weight normalized to body weight, an indicator of pulmonary edema, 1.30 ± 0.28 in cKO vs. 1.82 ± 0.25 in WT; Figure 1B), suggesting decreased pulmonary inflammation and edema. Consistently, viral nucleoprotein (NP) gene expression in lung homogenates was significantly reduced in cKO mice, indicating impaired viral replication (Figure 1C).

Histopathological analysis further supported these findings. Lung sections from WT mice showed severe pneumonia characterized by alveolar wall thickening, pulmonary edema, vascular congestion, and extensive inflammatory cell infiltration. In contrast, lung tissues from cKO mice displayed substantially milder pathological changes (Figure 1D). Consistent with the reduced viral replication and pathology, SNX10 deficiency was associated with an altered local inflammatory response. Cytokine profiling of lung tissue revealed significantly decreased levels of the pro-inflammatory cytokine TNF-α, accompanied by increased expression of the anti-inflammatory cytokine IL-10 and the antiviral cytokine IFN-γ (Figure 1E). This anti-inflammatory shift was further supported by elevated serum levels of IFN-γ in cKO mice compared to WT (Figure 1F).

Collectively, these results demonstrate that epithelial-specific SNX10 deficiency confers enhanced resistance to IAV infection by reducing viral replication, alleviating lung pathology, and modulating the host inflammatory response, ultimately leading to prolonged survival. Although SNX10 deficiency was associated with increased IFN-γ and IL-10, reduced TNF-α, and decreased viral load, our current data cannot distinguish whether this cytokine profile contributes to viral control or merely reflects reduced inflammatory stimulation. It is plausible that both mechanisms coexist: enhanced IFN-γ may directly suppress viral replication, while reduced viral burden subsequently limits TNF-α-mediated immunopathology. Future studies using cytokine neutralization or adoptive transfer in SNX10-deficient mice will be necessary to establish causal relationships.

### 3.2. SNX10 Is Essential for IAV Replication In Vitro

**Figure 2:**
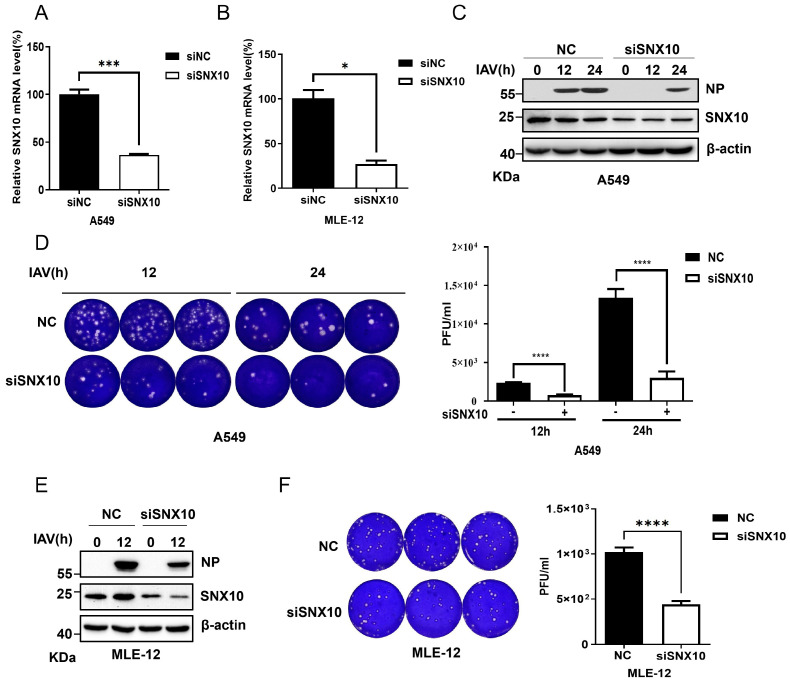
SNX10 knockdown inhibited influenza virus infection. (**A**,**B**) qRT-PCR analysis to verify the knockdown efficiency of siRNA in A549 (**A**) or MLE-12 cells (**B**) at 48 h post siRNA transfection; (**C**) Western blot analysis of NP expression in A549 cells transfected with siSNX10 or siNC and infected with PR8 (MOI = 0.1) for 12 and 24 h. (**D**) Plaque assay to measure viral titers in supernatants from A549 cells transfected with siSNX10 or siNC at 12 h and 24 h post-infection with PR8 (MOI = 0.1). (**E**) Western blot analysis of NP expression in MLE-12 cells transfected with siSNX10 or siNC and infected with PR8 (MOI = 0.1) for 12 h. (**F**) Plaque assay to measure viral titers in supernatants from MLE-12 cells transfected with siSNX10 or siNC at 12 h post-infection with PR8 (MOI = 0.1). Data are presented as the mean ± SD of three independent experiments and analyzed by two-tailed Student’s *t*-test (* *p* < 0.05, *** *p* < 0.001, **** *p* < 0.0001).

To further validate the regulatory role of SNX10 in IAV infection, we performed siRNA-mediated knockdown of endogenous SNX10 in human (A549) and murine (MLE-12) cells. The knockdown efficiency was confirmed by qRT-PCR (Figure 2A,B). Following transfection with either SNX10-targeting siRNA (siSNX10) or a non-targeting negative control (siNC), cells were infected with the PR8 strain of IAV at a multiplicity of infection (MOI) of 0.1. Viral replication was assessed at 12 h and 24 h post-infection. In SNX10-depleted A549 cells, viral NP expression was significantly reduced compared to controls (Figure 2C), accompanied by lower viral titers in the culture supernatants, as determined by plaque assay (Figure 2D). A similar reduction in NP expression and infectious virion production was observed in MLE-12 cells subjected to SNX10 knockdown (Figure 2E,F). Consistent with these findings, SNX10 silencing also suppressed NP expression of the WSN strain in A549 cells (Figure S1B), suggesting that the antiviral effect of SNX10 depletion is not strain-specific.

**Figure 3:**
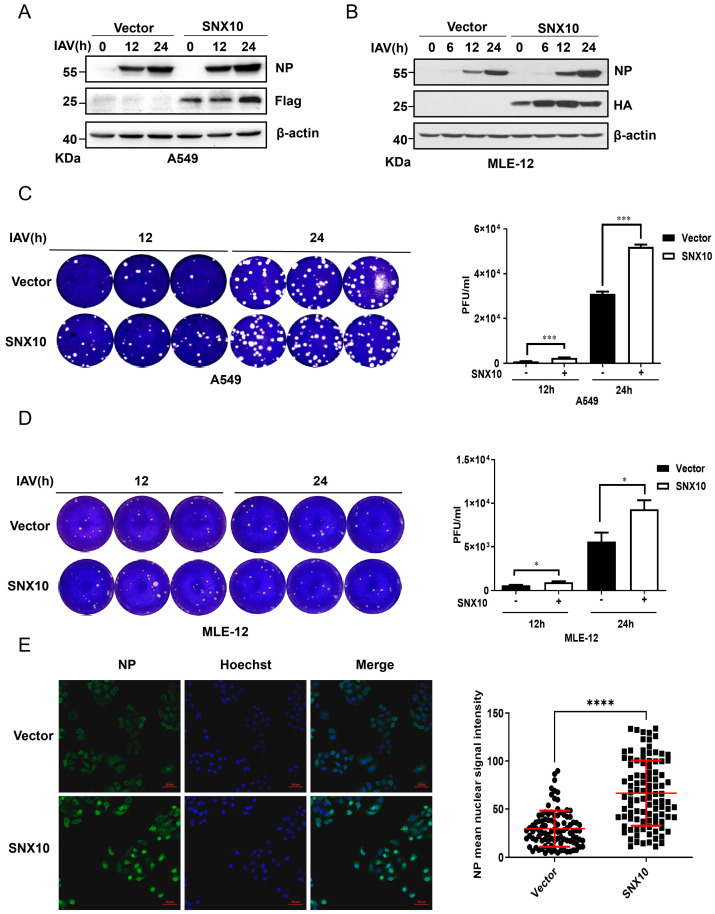
SNX10 overexpression promoted IAV infection. (**A**,**B**) Western blot analysis of NP expression in SNX10-overexpressing A549 cells (**A**) and MLE-12 cells (**B**) infected with PR8 (MOI = 0.1) for 12 and 24 h. (**C**,**D**) Plaque assay to measure viral titers in supernatants from SNX10-overexpressing A549 cells (**C**) and MLE-12 cells (**D**) at 12 and 24 h post-infection with PR8 at an MOI of 0.1. (**E**) Confocal immunofluorescence imaging of NP expression in SNX10-overexpressing A549 cells at 3 h post-infection with PR8 (MOI = 10). Cells were stained with NP antibody to label NP protein (green) and DAPI to label the nucleus (blue), Scale bar = 50 μm. Data are presented as the mean ± SD of three independent experiments and analyzed by two-tailed Student’s *t*-test (* *p* < 0.05, *** *p* < 0.001, **** *p* < 0.0001).

Conversely, overexpression of SNX10 in both A549 and MLE-12 cells led to a marked increase in viral NP protein levels (Figure 3A,B), as well as elevated viral titers in the supernatants (Figure 3C,D). Immunofluorescence staining further confirmed the enhancement of NP expression in SNX10-overexpressing A549 cells (Figure 3E). This pro-viral effect was similarly observed in another IAV subtype WSN infected cells overexpressing SNX10 (Figure S1C), reinforcing the role of SNX10 as a positive regulator of IAV replication.

### 3.3. SNX10 Mediated Acidic Vesicles Facilitates Viral Infection

Building on previous evidence that SNX10 interacts with the v-ATPase to promote the formation of acidic intracellular vesicles [18,22], and given the critical role of v-ATPase-mediated acidification in late endosomal viral uncoating [27,28], we hypothesized that SNX10 may modulate IAV infection through regulation of endosomal acidification.

**Figure 4:**
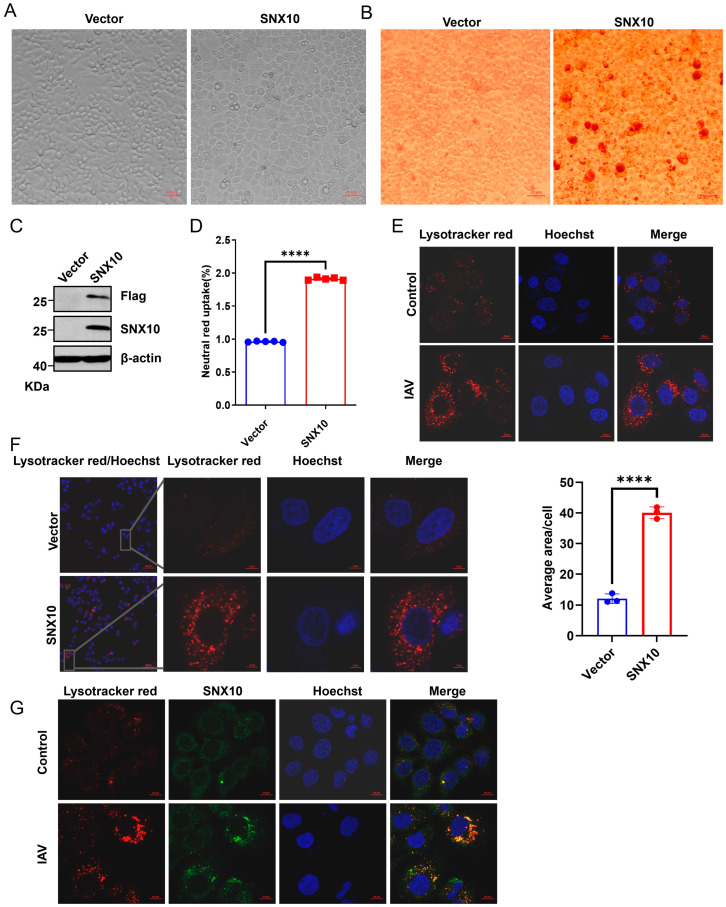
SNX10 induced acidic vacuoles to facilitate viral infection in cells. (**A**) Representative microscopy images showing cytoplasmic vacuolization in A549 cells overexpressing SNX10-Flag. (**B**) Neutral Red staining to detect acidic vacuoles in SNX10-overexpressing A549 cells (Scale bar = 20 μm); (**C**) Western blot analysis of SNX10-Flag expression in A549 cells. (**D**) Neutral Red uptake analysis of acidic vacuoles in SNX10-overexpressing A549 cells. (**E**) Confocal fluorescence microscopy of acidic vacuoles in A549 cells following infection with PR8 (MOI = 10) for 1 h. Cells were stained with LysoTracker Red to label acidic vacuoles (red) and DAPI to label the nucleus (blue), Scale bar = 10 μm (**left**) and quantitative analysis of LysoTracker Red intensity (**right**). (**F**) Confocal fluorescence microscopy of acidic vacuoles in SNX10-overexpressing A549 cells. Cells were stained with LysoTracker Red to label acidic vacuoles (red) and DAPI to label the nucleus (blue), Scale bar = 5 μm. (**G**) Confocal immunofluorescence imaging showing co-localization of SNX10 with acidic endosomes in A549 cells following infection with PR8 (MOI = 10) for 1 h. Cells were stained with LysoTracker Red to label acidic vacuoles (red), SNX10 antibody to label SNX10 protein (green), and DAPI to label the nucleus (blue), Scale bar = 10 μm. Data are presented as the mean ± SD of three independent experiments and analyzed by two-tailed Student’s *t*-test (**** *p* < 0.0001).

We constructed A549 cells stably overexpressing SNX10-Flag, and the overexpression efficiency was verified by Western blotting (Figure 4C). Notably, we observed that overexpression of SNX10 in A549 induced prominent cytoplasmic vacuolization, readily visible under light microscopy (Figure 4A). These vacuoles stained positively with the acidophilic dye Neutral Red (Figure 4B,D), indicating an acidic environment. Also, these acidic vesicles were confirmed using the lysosomal pH sensor LysoTracker™ Deep Red, which robustly labeled SNX10-induced vacuoles (Figure 4F and Figure S2A). During IAV infection, time-course imaging revealed progressive accumulation of acidic vesicles in infected cells (Figure 4E and Figure S3). Strikingly, confocal microscopy demonstrated precise colocalization of SNX10 with virus-induced acidic vesicles (Figure 4G), strongly suggesting that SNX10 contributes to the formation or maintenance of these compartments during infection.

**Figure 5:**
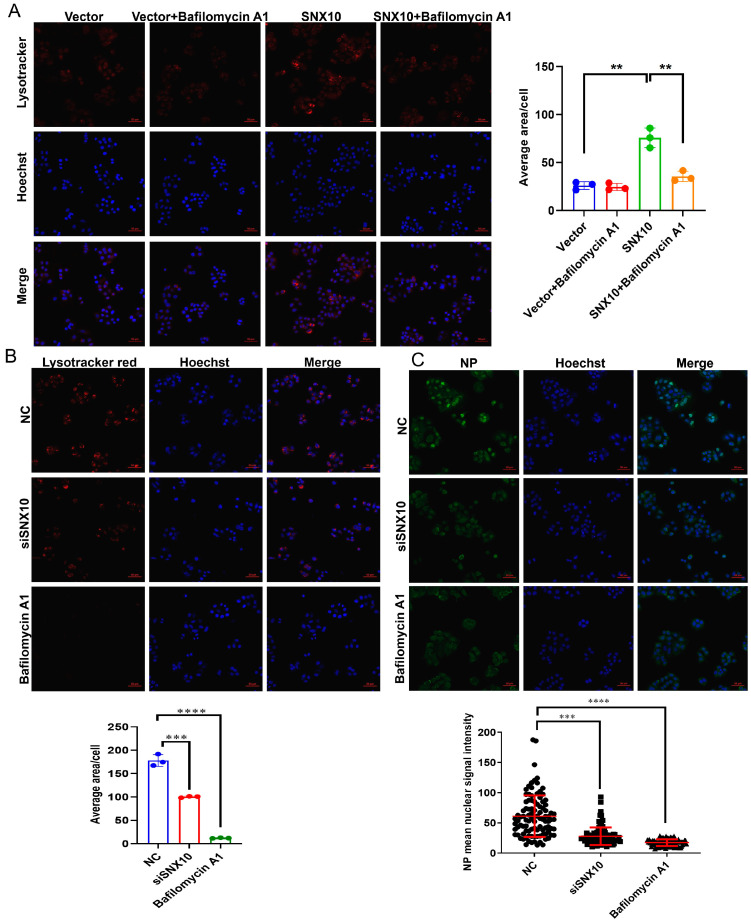
SNX10 was involved in the acidic endosome required for influenza virus entry. (**A**) Confocal fluorescence microscopy of acidic vacuoles in SNX10-overexpressing A549 cells following treatment with 10 nM Bafilomycin A1. Cells were stained with LysoTracker Red to label acidic vacuoles (red) and DAPI to label the nucleus (blue), Scale bar = 50 μm. (**B**) Confocal fluorescence microscopy of acidic vacuoles in A549 cells transfected with siSNX10 or treated with 10 nM Bafilomycin A1, followed by infection with PR8 (MOI = 10) for 1 h. Cells were stained with LysoTracker Red to label acidic vacuoles (red) and DAPI to label the nucleus (blue), Scale bar = 50 μm. (**C**) Confocal immunofluorescence imaging of NP expression in A549 cells transfected with siSNX10 or treated with 10 nM Bafilomycin A1, followed by infection with PR8 (MOI = 10) for 4 h. Cells were stained with NP antibody to label NP protein (green) and DAPI to label the nucleus (blue), Scale bar = 50 μm. Data are presented as the mean ± SD of three independent experiments and analyzed by two-tailed Student’s *t*-test (** *p* < 0.01, *** *p* < 0.001, **** *p* < 0.0001).

**Figure 6:**
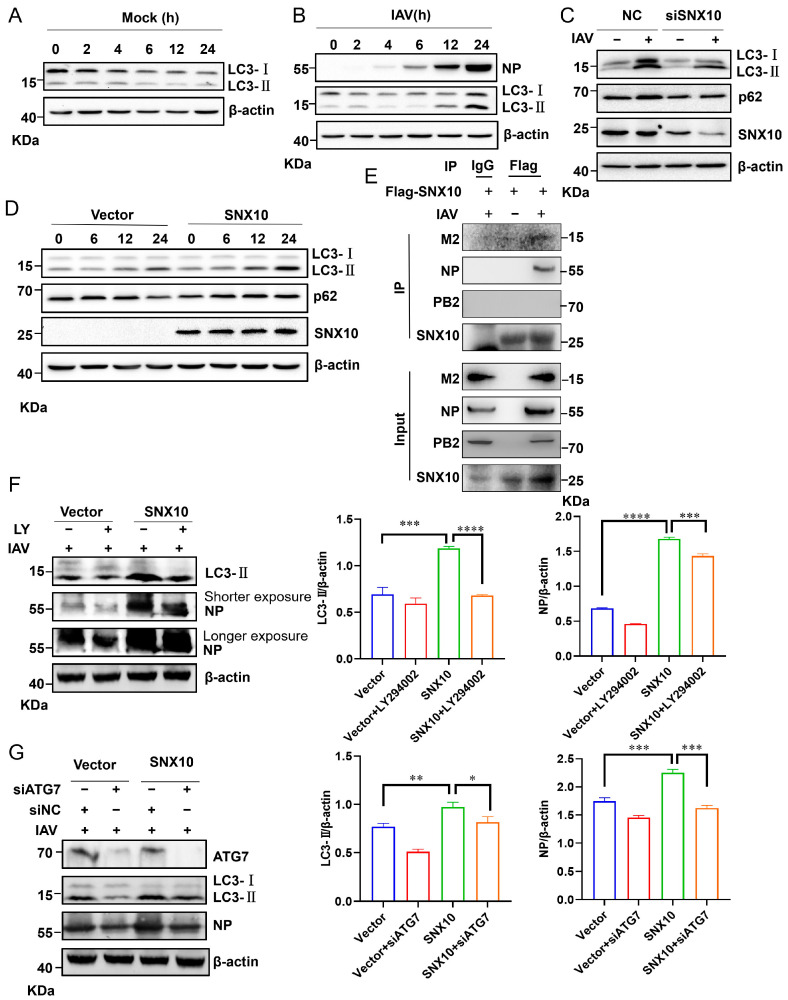
SNX10 induced IAV infection-associated autophagy through interaction with IAV M2 and NP protein. (**A**,**B**) Western blot analysis of LC3-I and LC3-II protein levels in A549 cells infected with PR8 (MOI = 1) for the indicated durations. (**C**) Western blot analysis of LC3-I and LC3-II protein levels in A549 cells transfected with siSNX10 or siNC and infected with PR8 (MOI = 1) for 24 h. (**D**) Western blot analysis of LC3-I and LC3-II protein levels in SNX10-overexpressing A549 cells infected with PR8 (MOI = 1). (**E**) Co-immunoprecipitation was used to detect the interaction between SNX10 and IAV M2 and NP protein in A549 cells. (**F**) Western blot analysis of NP and LC3-II protein levels in the control and SNX10 overexpressed A549 cells infected with PR8 (MOI = 1) for 24 h in the presence of LY294002 (10 μM). (**G**) The siNC and siATG7 were transfected into the control and SNX10 overexpressed A549 cells, respectively, then the cells were infected with PR8 (MOI = 1), at 24 h post-infection, the LC3-II and NP proteins were detected. Data are presented as the mean ± SD of three independent experiments and analyzed by two-tailed Student’s *t*-test (* *p* < 0.05, ** *p* < 0.01, *** *p* < 0.001, **** *p* < 0.0001).

To determine whether v-ATPase activity is required for this process, we treated SNX10-overexpressing cells with the selective v-ATPase inhibitor bafilomycin A1 (10 nM). This treatment completely abrogated vacuole formation (Figure 5A and Figure S2B), validating the dependence of SNX10-induced vesicle acidification on v-ATPase activity. To investigate the role of endosomal acidification in SNX10-mediated pro-viral activity, we utilized bafilomycin A1 (10 nM), a specific inhibitor of v-ATPase. As expected, pre-treatment of cells with bafilomycin A1, which is known to block the critical low-pH step required for IAV membrane fusion during entry [29], drastically abrogated the formation of virus-induced vacuoles and subsequent viral NP expression (Figure 5B,C). It is acknowledged that bafilomycin A1 pre-treatment inhibits both endosomal acidification (required for viral entry) and autophagic flux (required for lysosomal degradation). Therefore, the reduced viral replication observed under this condition could be attributed to impaired viral entry rather than solely to blockade of autophagic degradation. To specifically address whether SNX10 promotes viral replication through the modulation of autophagy, we performed rescue experiments using ATG7 knockdown. ATG7 serves as a critical E1-like activating enzyme that facilitates the conjugation of ATG12 to ATG5 and the conversion of LC3-I to its lipidated form, LC3-II; both processes are essential for the nucleation and elongation of the autophagosome membrane [30,31]. Our results demonstrated that the depletion of ATG7 in SNX10-overexpressing cells effectively suppressed the elevation of viral NP protein levels typically induced by SNX10 (Figure 6G). The significant reduction in viral titer suggested that SNX10-mediated viral propagation was dependent on the functional integrity of the autophagy pathway, occurring independently of the viral entry stage.

### 3.4. SNX10 Exacerbates Pro-Viral Autophagy via Interaction with the IAV M2 Protein

Successful viral infection requires the efficient release of viral genomes following entry into host cells. While various receptors facilitate viral entry, the acidic intracellular compartments—such as endosomes and autolysosomes—serve as essential platforms for viral uncoating and trafficking [15]. Autophagy, a conserved cellular stress response pathway, is frequently hijacked by viruses to support infection and replication [32,33]. In line with this, IAV infection led to increased levels of LC3-II, a marker of autophagosome formation, indicating an accumulation of autophagic structures (Figure 6A,B). Strikingly, overexpression of SNX10 further amplified LC3-II accumulation following IAV infection, whereas SNX10 knockdown attenuated LC3-II levels (Figure 6C,D). Meanwhile, we detected the expression of the autophagic flux marker protein p62. Under conditions where SNX10 was knocked down, the decrease in LC3-II following viral infection was accompanied by downregulated p62 expression during IAV infection (Figure 6C), suggesting restoration of autophagic degradation. In wild-type cells, with prolonged viral infection, p62 expression decreased at 24 h. Consistently, in cells overexpressing SNX10, p62 expression increased at 24 h post-infection (Figure 6D). The simultaneous accumulation of LC3-II and p62 upon SNX10 overexpression is a classical signature of autophagic flux blockade, leading to the accumulation of non-functional autophagosomes. These findings indicate that SNX10 is a key regulator of cellular autophagic homeostasis under viral infection. Furthermore, we identified physical interactions between SNX10 and the viral M2 protein and NP protein during infection (Figure 6E), which was consistent with previous reports that M2 and NP induce autophagosome accumulation via AKT-mTOR signaling [15]. Also, we did not detect the interaction of SNX10 with PB2 protein during IAV infection. To further probe the functional relevance of autophagy in SNX10-mediated viral replication, we treated cells with the PI3K inhibitor LY294002, which blocks autophagy initiation. In both wild-type and SNX10-overexpressing cells, LY294002 treatment suppressed LC3-I lipidation to LC3-II and reduced NP expression (Figure 6F), supporting a pro-viral role for autophagy during IAV infection. Additionally, genetic ablation of ATG7—an E1-like enzyme essential for LC3 and ATG12 conjugation—markedly reduced LC3-II accumulation and impaired viral replication (Figure 6G). These results indicate that SNX10, relying on its interaction with M2 and NP proteins, promotes the accumulation of autophagic structures during IAV infection, and that this virus-induced accumulation of autophagic structures facilitates viral propagation.

### 3.5. IAV Infection Stabilizes SNX10 by Suppressing Ubiquitination-Dependent Degradation

**Figure 7:**
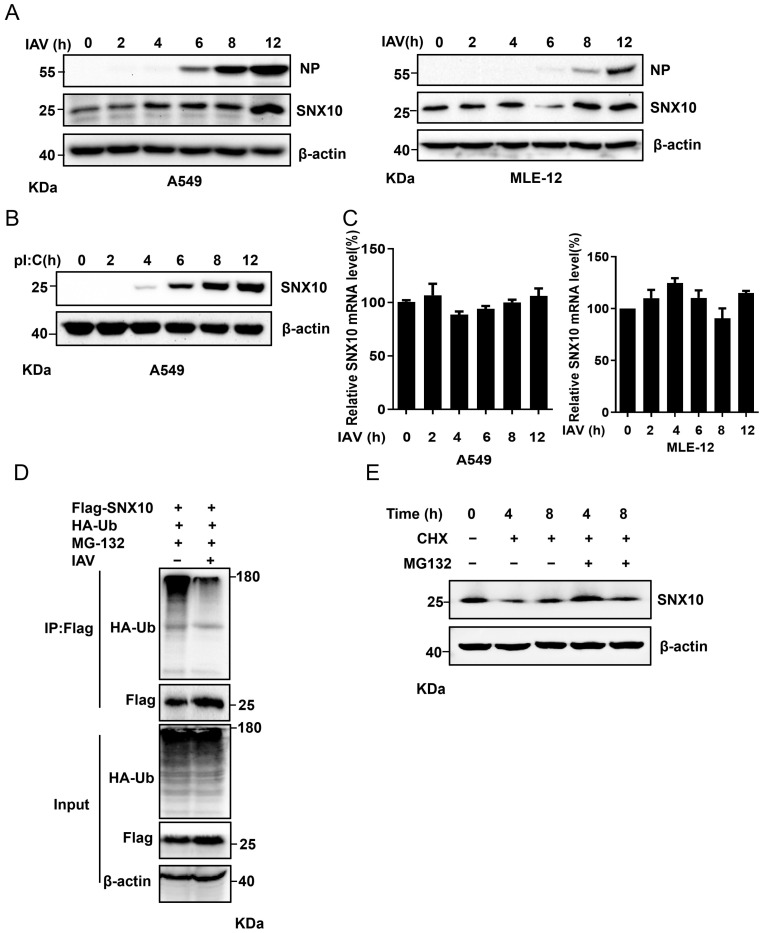
Influenza virus infection inhibited the degradation of SNX10 via the ubiquitin–proteasome pathway. (**A**) Western blot analysis of SNX10 expression in A549 cells (left) and MLE-12 cells (right) infected with PR8 (MOI = 1) for the indicated time. (**B**) Western blot analysis of SNX10 expression in A549 cells transfected with poly I:C (1 μg/mL) for the indicated time. (**C**) qRT-PCR analysis of SNX10 mRNA expression in A549 cells (**left**) and MLE12 cells (**right**) infected with PR8 (MOI = 1) for the indicated time. (**D**) Immunoprecipitation analysis of ubiquitinated SNX10 in A549 cells infected with PR8 (MOI = 1) for 20 h and treated with MG-132 (20 μM) for the final 4 h. (**E**) A549 cells were treated with CHX (100 μg/mL) for different times combined with or without MG132 (20 μM).

To investigate whether IAV affects the cellular abundance of SNX10, we infected A549 and MLE-12 cells with IAV and tested SNX10 expression at the protein and mRNA levels. The results indicated that SNX10 increased progressively during infection (Figure 7A), a trend mirrored in poly I:C-transfected A549 cells (Figure 7B). However, qRT-PCR analysis revealed no significant change in SNX10 mRNA levels following infection (Figure 7C), ruling out transcriptional regulation. Given that the ubiquitin–proteasome system (UPS) plays a major role in post-translational protein turnover, immunoprecipitation assays in IAV-infected A549 cells demonstrated a marked reduction in SNX10 ubiquitination compared with uninfected controls (Figure 7D). Furthermore, when the cells were treated with the protein synthesis inhibitor CHX, the CHX-induced SNX10 decrease could be blocked by the proteasome inhibitor MG132 (Figure 7E). Collectively, these findings indicate that IAV infection is associated with the stabilization of SNX10, correlating with reduced ubiquitination and subsequent proteasomal degradation—a strategy that promotes SNX10-dependent pro-viral processes. However, the cellular environment during viral infection is highly complex, involving various viral proteins and host responses. The regulation of SNX10 ubiquitination after viral infection still needs to be furtherly explored in the future.

## 4. Discussion

The interplay between IAV and host proteins critically determines viral pathogenicity [34]. A host factor of interest is SNX10, a member of the PX domain-containing SNX family that regulates endosomal trafficking and signaling [35,36]. Its PX domain aids in the targeting of SNX proteins to phosphoinositide-enriched endosomal membranes. Perturbations in SNX10 function are linked to human diseases, including osteopetrosis [37], colorectal cancer [38], and ovarian cancer [39], highlighting its physiological importance. Crucially, in the context of viral infection, SNX10 has been shown to activate v-ATPase to induce endosomal acidification [22], a process essential for viral entry, thus suggesting SNX10 as a plausible host factor in the IAV life cycle. Our previous study found that SNX10 enhances HCoV-OC43 infection by facilitating viral entry and inhibiting virus-triggered autophagy [25]. The critical role of SNX proteins in viral replication is further highlighted by other family members; for instance, Igor Štimac et al. reported that the SNX27-Retromer-ESCPE-1 pathway regulates endosomal tubule formation, a process exploited by human cytomegalovirus (HCMV) to facilitate its replication cycle [40]. Similarly, Tuğba Koçmar et al. found that SNX2 interacts with the IAV PA protein and negatively regulates virus replication [41]. These findings underscore a specific mechanism by which a member of the SNX family can regulate IAV infection.

In the present study, we initially observed that in vivo knockdown of SNX10 in mice prolonged survival and attenuated the inflammatory response induced by the virus. Subsequent in vitro experiments corroborated the hypothesis that SNX10 removal effectively reduced viral titers, while its exogenous overexpression significantly increased viral titers. This evidence suggests that SNX10 acts as a positive regulator of IAV infection. Given that SNX10 is known to regulate the intracellular acidic environment and promote the formation of acidic endosomes—conditions favorable for viral replication—we sought to determine which stage of the IAV life cycle it affects. The IAV life cycle, spanning approximately 8–10 h, comprises adsorption, entry, replication, and release [42]. We found that SNX10 colocalized with virus-induced acidic vesicles and these acidic vesicles could be abrogated by pre-treating with bafilomycin A1, a specific inhibitor of v-ATPase. These data indicated that SNX10 cooperated with the v-ATPase complex to promote the formation of acidic endosomes. This acidification is a prerequisite for HA-mediated membrane fusion, suggesting that SNX10 provides the essential low-pH environment required for efficient viral uncoating and genome release.

Autophagy, a lysosomal degradation pathway, plays essential roles in immunity, including immune system development, regulation of innate and adaptive immune and inflammatory responses, selective degradation of intracellular microbes, and host protection against infectious diseases. Accumulating research has also suggested that autophagy is a critical mechanism in host defense responses against IAV infection by degrading viral particles and activating innate or acquired immunity to induce viral clearance. However, IAV has conversely hijacked autophagy to strengthen viral infection by blocking autophagy maturation and further interfering with host antiviral signaling to promote viral replication. Therefore, how the battle for autophagy between host and IAV is carried out needs to be known. Previous studies revealed that alteration of autophagy significantly affects the early stages of the influenza virus life cycle or viral RNA synthesis; at the same time, both IAV M2 and NP colocalize and interact with LC3 puncta mediating the AKT-mTOR-dependent autophagy in host cells, leading to an increase in viral ribonucleoprotein (vRNP) export and infectious viral particle formation, indicating that the IAV-host autophagy interaction plays a critical role in regulating IAV replication [15]. In our study, we found that SNX10 overexpression elevated LC3-II levels and increased p62 accumulation following viral infection, consistent with an inhibition of autophagic flux. Conversely, SNX10 knockdown reduced LC3-II expression along with decreased p62 levels, suggesting restoration of autophagic degradation. The simultaneous accumulation of LC3-II and p62 upon SNX10 overexpression is a classical signature of autophagic flux blockade, leading to the accumulation of non-functional autophagosomes. Interestingly, we detected that SNX10 interacts with both the IAV M2 and NP. Given M2 and NP’s known role in autophasome induction via the Akt/mTOR pathway, we investigated this link. We found that inhibiting autophagy (via siATG7 transfection) or the PI3K/Akt pathway (via LY294002) attenuated LC3-II accumulation and reduced NP expression. Furthermore, as M2 is known to disrupt the TBC1D5-Rab7 interaction to prevent lysosomal degradation of viral components [16].

Based on our findings and existing literature, we propose a mechanistic model that may explain the observed co-existence of increased acidic vesicles alongside inhibited autophagic flux upon SNX10 overexpression during viral infection. The IAV M2 (a proton channel critical for endosomal acidification) and NP, were found to interact with SNX10. Given that NP has been found as an autophagy inducer to promote IAV replication and M2 has also been implicated in blocked autophagosome fusion with lysosomes [15,43], we speculate that the SNX10-NP and SNX10-M2 interactions promote the accumulation of non-functional autophagosomes. We hypothesize that the SNX10-NP interaction induces autophagy and the SNX10-M2 interaction leads to the aberrant recruitment or retention of the M2 proton channel on phagophore or early autophagosomal membranes. This results in the premature and direct acidification of the autophagosome lumen via M2’s ion channel activity, independent of lysosomal fusion. Such M2-mediated acidification, coupled with potential SNX10-mediated alterations in membrane lipid composition, likely disrupts the normal maturation cascade—particularly the recruitment of fusion machinery (e.g., specific RABs or SNAREs). Consequently, we speculated that by physically interacting with NP and M2, SNX10 facilitates the initial formation of autophagosomes (via NP) but subsequently prevents their maturation into autolysosomes (via M2). This led to the accumulation of non-functional autophagosomes. These autophagosomes may exhibit an acidic interior (consistent with increased acidic vesicles) but possess impaired fusion with lysosomes, leading to a blockade in autophagic flux and the accumulation of LC3-II and p62. These modified, non-degradative autophagic compartments may serve as a “viral-friendly niche” that shelters viral components from degradation, provides a membrane platform for replication, and concurrently depletes cellular resources by stalling functional autophagy, thereby creating a favorable environment for viral replication.

Furthermore, our results demonstrated that IAV stimulation increased SNX10 protein expression without significantly altering its mRNA levels, suggesting post-transcriptional regulation. Mechanistically, our findings indicated that IAV infection correlated with reduced SNX10 ubiquitination, leading to its stabilization. Consistent with the pro-viral role of SNX10-mediated autophagy, inhibiting autophagy in host cells effectively counteracted the increased viral replication caused by SNX10 overexpression. This aligns with broader evidence implicating SNX proteins in virus–autophagy interplay. For example, Dong et al. suggested that SNX5 localizes to virion-containing endosomes to initiate autophagy via the ATG14-PI3KC3-C1 complex, yet its deletion unexpectedly increased susceptibility to viral infection [44]. These contrasting findings highlight the complex and context-dependent roles of SNX proteins in viral infections and underscore the need for further investigation into the SNX family’s regulation of autophagy during viral infection.

The opposing effects of SNX family members on IAV infection—with SNX2 and SNX5 suppressing while SNX10 promoting viral replication—raise an intriguing question regarding their structural and functional differences. SNX2 and SNX5 belong to the SNX-BAR subfamily, characterized by an N-terminal PX domain that binds phosphoinositides and a C-terminal BAR (Bin/Amphiphysin/Rvs) domain that forms a banana-shaped dimer capable of sensing and inducing membrane curvature [45]. This structural feature enables SNX-BAR proteins to regulate membrane tubulation, membrane deformation, and retromer-mediated sorting [45]. In contrast, SNX10 belongs to the SNX-PX subfamily, which contains only the PX domain without additional conserved domains such as BAR or FERM [46]. SNX-PX proteins are primarily involved in endosomal signaling and trafficking regulation through protein–protein interactions. SNX10 specifically interacts with the v-ATPase complex to promote endosomal acidification [22]. These distinct structural features likely explain the divergent roles of SNX proteins in IAV infection: SNX-BAR proteins utilize their membrane remodeling capacity to enhance endosomal sorting and degradation of viral components, while SNX10 promotes endosomal acidification and autophagosome accumulation that benefit viral replication.

## 5. Conclusions

**Figure 8:**
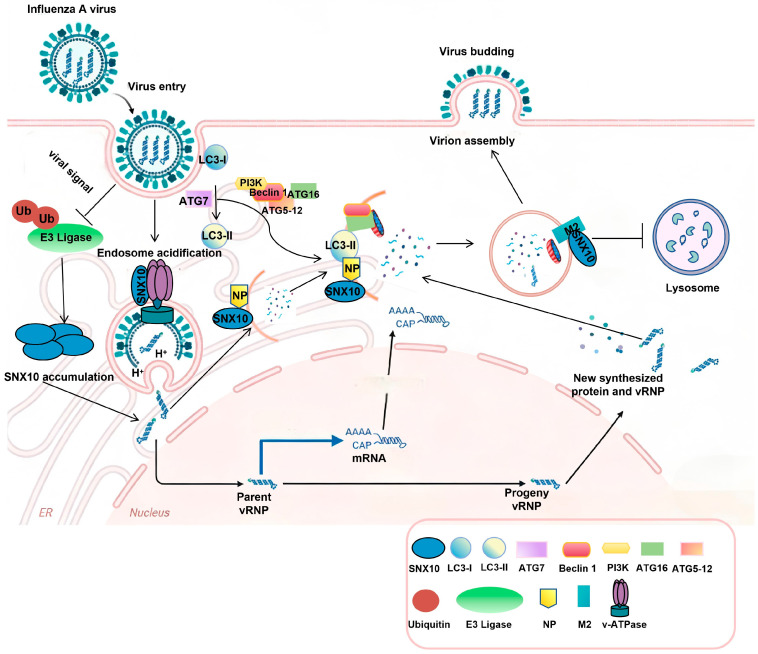
Proposed model of the dual role of SNX10 in the Influenza A Virus (IAV) life cycle. SNX10 acts as a critical host dependency factor during two distinct stages of infection. (Stage 1: Viral Entry) During the early phase of infection, SNX10 colocalizes with endosomal markers and cooperates with the v-ATPase complex to facilitate endosomal acidification. This low-pH environment is essential for HA-mediated membrane fusion and successful viral uncoating. Pre-treatment with Bafilomycin A1 or SNX10 deficiency disrupts this acidification, leading to a blockade of viral entry. (Stage 2: Viral Replication and Autophagic Subversion) In the later stages of infection, IAV stabilizes SNX10 by inhibiting its ubiquitination. Increased SNX10 levels provide a molecular scaffold that interacts with both viral NP and M2 proteins. This interaction initiates the formation of autophagosomes but prevents their fusion with lysosomes, resulting in the accumulation of non-functional autophagosomes (marked by elevated LC3-II and p62). These arrested vesicles serve as protected platforms that favor viral assembly and progeny production while avoiding lysosomal degradation.

In conclusion, our study identified IAV infection actively enhanced SNX10 protein levels during the course of infection, which was accompanied by a marked decrease in SNX10 ubiquitination, suggesting a deliberate “hijacking” of the host’s protein quality control machinery. The stabilization of SNX10 had immediate consequences for the early stages of the viral life cycle. Our data demonstrated that SNX10 colocalized with virus-induced acidic vesicles and cooperated with the v-ATPase complex. By promoting the formation of acidic endosomes, SNX10 provided the low-pH environment required for HA-mediated membrane fusion. Beyond entry, we found that SNX10 played a profound role in modulating IAV-induced autophagy. The observation that SNX10 overexpression led to simultaneous elevation of LC3-II (a marker of autophagosome formation) and p62/SQSTM1 (a marker of impaired degradation) strongly indicated a blockade of autophagic flux. We propose that SNX10 achieves this blockade through direct physical interaction with the viral proteins NP and M2. While NP is a known inducer of autophagy, the M2 protein contains a highly conserved LC3-interacting region (LIR) that halts autophagosome-lysosome fusion. SNX10 likely acts as a molecular bridge, stabilizing the interaction between these viral components and the host’s autophagic machinery. By stalling the flux, SNX10 and IAV ensure that LC3-positive membranes are not consumed by lysosomes but are instead diverted to support viral ribonucleoprotein (vRNP) transport or filamentous budding at the plasma membrane (Figure 8). These findings establish SNX10 as a promising host target for developing novel anti-influenza strategies.

## Data Availability

The data that support the findings of this study are available from the corresponding author upon reasonable request.

## Supplementary Materials

The following supporting information can be downloaded at: https://www.mdpi.com/article/10.3390/v18040460/s1. Figure S1: SNX10 modulates IAV replication in vitro and in vivo. (A) The expression of SNX10 protein in WT and SNX10 cKO mice lung homogenates. (B) The expression of NP protein in A549 cell after the cells were transfected with siNC and siSNX10 and infected with WSN at MOI = 0.1 for 24 h. (C) The expression of NP in control A549 cell and SNX10 exogenous cell. Figure S2: SNX10 overexpression and IAV infection modulate the formation of acidic vacuoles. (A) Acidic vacuoles induced by overexpressing SNX10 in cells were detected by Lysotracker red. (B) The IAV infection-induced vacuoles could be inhibited by bafilomycin A1. Figure S3: Time-course analysis of acidic vacuoles formation following IAV infection in A549 cells. Cells were infected with PR8 at 10 MOI and stained with LysoTracker Red DND-99 at a final concentration of 50 nM; the acidic vesicle formation was detected at different time points.

## Abbreviations

The following abbreviations are used in this manuscript:

IAV	Influenza A Virus
SNX10	Sorting Nexin 10
HA	hemagglutinin
NP	nucleoprotein
M2	matrix protein 2
v-ATPase	vacuolar-type H^+^-ATPase
MDCK	Madin-Darby canine kidney
MLE-12	mouse lung epithelial cells
DMEM	Dulbecco’s Modified Eagle Medium
cKO	conditional knockout
WT	wild-type
qRT-PCR	quantitative reverse transcriptase- PCR
CHX	Cycloheximide
MOI	multiplicity of infection
