# Supplementary material for: Sorting Nexin 10 Mediates Endosomal Acidification and Autophagy to Promote Influenza A Virus Infection
Source: Viruses. 2026 Apr 12;18(4):460. doi: 10.3390/v18040460 (PMC13119817; doi:10.3390/v18040460)
Supplement: Supplementary file 1 [file viruses-18-00460-s001.zip › viruses-4136532-supplementary.pdf]

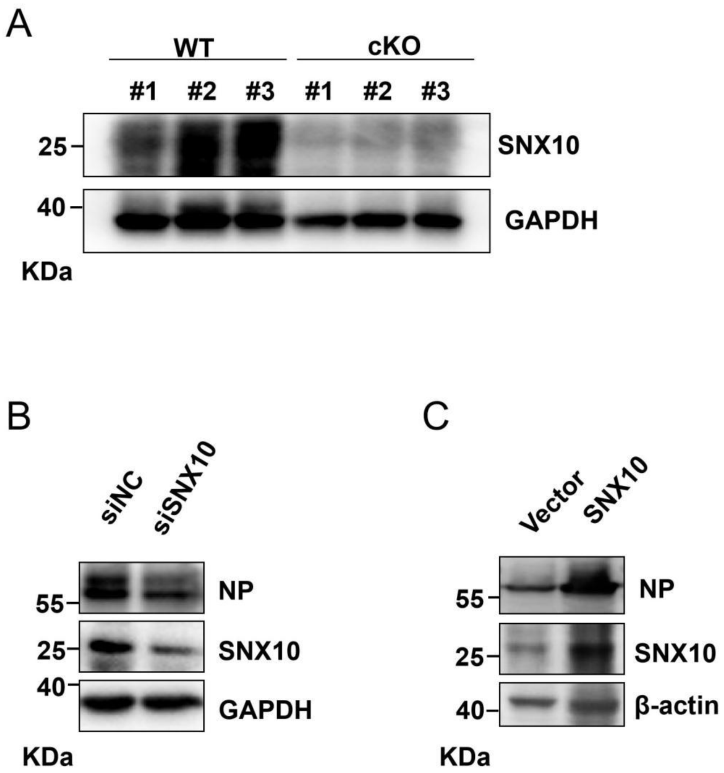

Figure S1. SNX10 modulates IAV replication in vitro and in vivo. (A) The expression of SNX10 protein in WT and SNX10 cKO mice lung homogenates. (B) The expression of NP protein in A549 cell after the cells were transfected with siNC and siSNX10 and infected with WSN at MOI = 0.1 for 24 h. (C) The expression of NP in control A549 cell and SNX10 exogenous cell.

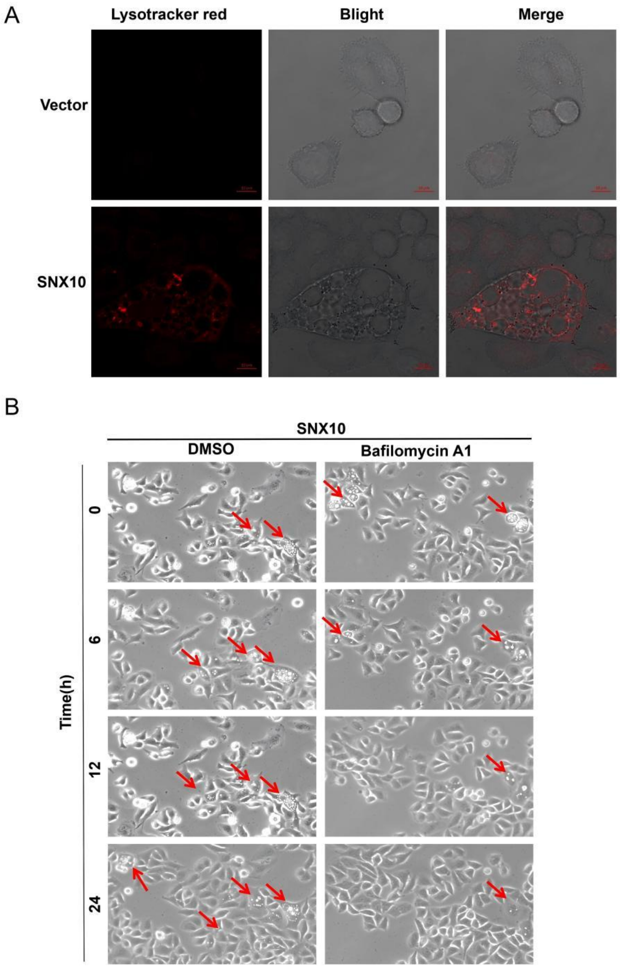

Figure S2. SNX10 overexpression and IAV infection modulate the formation of acidic vacuoles. (A) Acidic vacuoles induced by overexpressing SNX10 in cells were detected by Lysotracker red. (B) The IAV infection-induced vacuoles could be inhibited by bafilomycin A1.

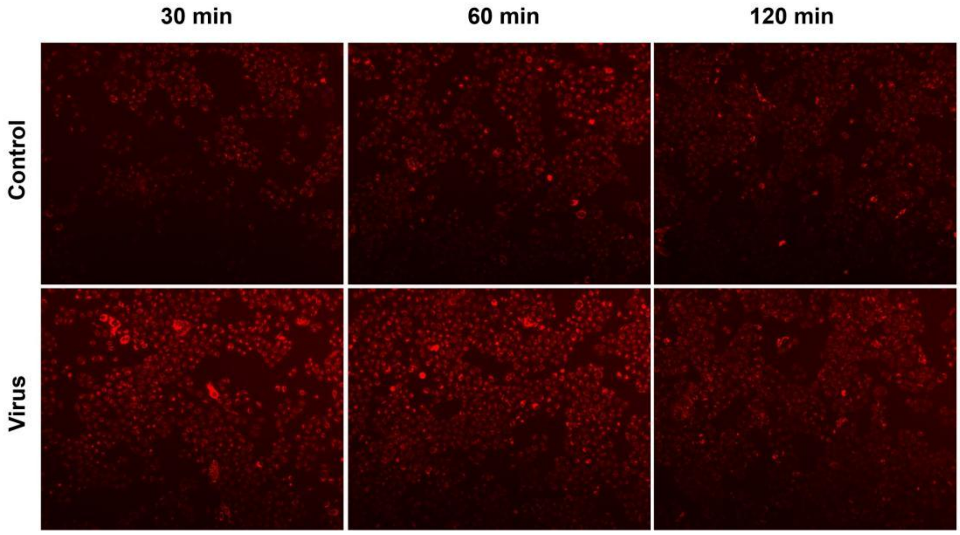

Figure S3. Time-course analysis of acidic vacuoles formation following IAV infection in A549 cells. Cells were infected with PR8 at 10 MOI and stained with LysoTracker Red DND-99 at a final concentration of 50 nM; the acidic vesicle formation was detected at different time points.
